# Improved 6‐year overall survival in AT/RT – results of the registry study Rhabdoid 2007

**DOI:** 10.1002/cam4.741

**Published:** 2016-05-26

**Authors:** Kerstin Bartelheim, Karolina Nemes, Angela Seeringer, Kornelius Kerl, Jochen Buechner, Joachim Boos, Norbert Graf, Matthias Dürken, Joachim Gerss, Martin Hasselblatt, Rolf‐Dieter Kortmann, Irene Teichert von Luettichau, Inga Nagel, Randi Nygaard, Florian Oyen, Eduardo Quiroga, Paul‐Gerhardt Schlegel, Irene Schmid, Reinhard Schneppenheim, Reiner Siebert, Palma Solano‐Paez, Beate Timmermann, Monika Warmuth‐Metz, Michael Christoph Frühwald

**Affiliations:** ^1^ Children's Hospital AugsburgSwabian Children's Cancer CenterStenglinstr. 286156AugsburgGermany; ^2^ Department of Pediatric Hematology and OncologyUniversity Children's Hospital MünsterAlbert‐Schweitzer‐Str. 3348149MünsterGermany; ^3^ Department of PediatricsUniversity Hospital of North‐NorwayTromsøNorway; ^4^ Department of Pediatric MedicineOslo University Hospital RikshospitaletP.O. Box 4950 NydalenN‐0424OsloNorway; ^5^ Department of Pediatric Hematology and OncologyUniversity of SaarlandGebäude 966421HomburgGermany; ^6^ Department of Pediatric Hematology and OncologyUniversity MannheimTheodor‐Kutzer‐Ufer 1‐368167MannheimGermany; ^7^ Institute of Biostatistics and Clinical ResearchUniversity of MünsterSchmeddingstraße 5648149MünsterGermany; ^8^ Institute of NeuropathologyUniversity Hospital MünsterPottkamp 248149MünsterGermany; ^9^ Department of RadiooncologyUniversity of LeipzigStephanstraße 9a04103LeipzigGermany; ^10^ Children's Hospital Medical CenterTechnische UniversitätMunich, Kölner Platz 180804MünchenGermany; ^11^ Institute of Human GeneticsChristian‐Albrechts‐University Kiel & University Hospital Schleswig‐ HolsteinCampus Kiel, Arnold‐Heiler‐Str. 324105KielGermany; ^12^ Department for Children and AdolescentsSection for Pediatric Hematology/OncologySt Olav's HospitalUniversity Hospital of Trondheim7006TrondheimNorway; ^13^ Department of Pediatric Hematology and OncologyUniversity Medical Center Hamburg‐EppendorfMartinistraße 5220246HamburgGermany; ^14^ Department of Pediatric OncologyHospital Infantil Virgen del RocioAVDA Manuel Siurot S/N41013SevillaSpain; ^15^ Department of Pediatric Hematology and OncologyUniversity WürzburgJosef‐Schneider‐Str. 297080WürzburgGermany; ^16^ Department of Pediatric Hematology and OncologyLudwig‐Maximilian‐University MunichLindwurmstr. 480337MünchenGermany; ^17^ Particle Therapy Clinic at West German Proton TherapyUniversity Hospital EssenHufelandstr. 5545147EssenGermany; ^18^ Department of NeuroradiologyUniversity Hospital WürzburgJosef‐Schneider‐Str. 1197080WürzburgGermany

**Keywords:** AT/RT, EU‐RHAB Registry, pediatric brain tumor, Rhabdoid 2007

## Abstract

Atypical teratoid rhabdoid tumors (AT/RT) are characterized by mutations and subsequent inactivation of *SMARCB1* (*INI1, hSNF5*), a predilection for very young children and an unfavorable outcome. The European Registry for rhabdoid tumors (EU‐RHAB) was established to generate a common European database and to establish a standardized treatment regimen as the basis for phase I/II trials. Thus, genetic analyses, neuropathologic and radiologic diagnoses, and a consensus treatment regimen were prospectively evaluated. From 2005 to 2009, 31 patients with AT/RT from four countries were recruited into the registry study *Rhabdoid 2007* and treated with systemic and intraventricular chemotherapy. Eight patients received high‐dose chemotherapy, 23 radiotherapy, and 17 maintenance therapy. Reference evaluations were performed in 64% (genetic analyses, FISH, MLPA, sequencing) up to 97% (neuropathology, INI1 stain). Germ‐line mutations (GLM) were detected in 6/21 patients. Prolonged overall survival was associated with age above 3 years, radiotherapy and achievement of a complete remission. 6‐year overall and event‐free survival rates were 46% (±0.10) and 45% (±0.09), respectively. Serious adverse events and one treatment‐related death due to insufficiency of a ventriculo peritoneal shunt (VP‐shunt) and consecutive herniation were noted. Acquisition of standardized data including reference diagnosis and a standard treatment schedule improved data quality along with a survival benefit. Treatment was feasible with significant but manageable toxicity. Although our analysis is biased due to heterogeneous adherence to therapy, EU‐RHAB provides the best available basis for phase I/II clinical trials.

## Introduction

The development of specific diagnostic measures including immunohistochemistry and molecular genetics has led to a precise definition of the entity rhabdoid tumors 1. The majority of rhabdoid tumors demonstrate mutations in *SMARCB1* (*INI1, hSNF5*) 2, 3 and rarely *SMARCA4* (*BRG1*) 4. Survival remains unsatisfactory due to the aggressive nature of the disease and ranges from 15% to 40% even with intensive multimodality therapy 5, 6. Dufour et al. report 58 nonuniformly treated patients with atypical teratoid rhabdoid tumor (AT/RT, 1998–2008) with a median overall survival (OS) of 9 months 7. Chi et al. demonstrated a promising 2‐year OS of 70% treated in a prospective protocol 8.

The implementation of the European registry for rhabdoid tumors (EU‐RHAB) – was intended to prospectively collect comprehensive data on consistently treated children with AT/RT across several European countries. Data collection was initiated in 2005 with centers mainly from Germany and Austria but also Scandinavia and Spain. The registry provides basic clinical data and a system of high‐quality reference diagnostics and expert counseling for diagnostic and therapeutic measures. Thus, EU‐RHAB was an innovation in AT/RT research at the time of initiation despite the fact that the quality of resulting data may not entirely be comparable to a controlled clinical trial. Children with rare diseases and a risk profile, which makes a controlled clinical trial difficult, have the same rights for a standardized treatment with the best available scientific evidence of activity. The treatment schedule *Rhabdoid 2007*, specifically designed for rhabdoid tumors, comprised elements including anthracyclines, intraventricular methotrexate and early start of radiotherapy (RT), all of which have previously been described as promising tools in this entity 6, 9.

## Materials and Methods

From 06/2005 to 03/2009 a total of 35 patients with a centrally confirmed diagnosis of AT/RT were registered to EU‐RHAB. Thereof 31 patients were treated according to the *Rhabdoid 2007* schedule. Eight patients received high‐dose chemotherapy (HDCT) with autologous stem cell transplantation following induction chemotherapy at the treating physicians discretion. Results for these eight patients have partly been reported 10. Inclusion criteria were diagnosis of an AT/RT as confirmed by negative INI1 staining and typical neuropathological features as confirmed by a reference neuropathologist, age below 18 years of age and the presence of informed consent by the legal guardians to contribute patient‐associated data.

EU‐RHAB including *Rhabdoid 2007* has received continuous approval by the Ethics Committee of the University of Münster (ID 2009‐532‐f‐S).

### Diagnostic measures

Reference neuropathology including immunohistochemistry of SMARCB1 was performed according to WHO criteria on formalin‐fixed paraffin‐embedded (FFPE) tumor tissue 1. Staging procedures such as evaluation of lumbar cerebrospinal fluid (CSF) was in accordance with the Chang classification 11. Neuroradiologic response was evaluated according to criteria of the German National Reference Center for Neuroradiology in Würzburg, Germany 12. DNA was derived from FFPE tumor material and peripheral blood cells, and analyzed for somatic and germ‐line genetic changes in *SMARCB1* (*SMARCA4*) to identify tumor associated and constitutional aberrations. Fluorescence‐in situ hybridization (FISH), multiplex ligation‐dependent probe amplification (MLPA), and sequencing were performed at reference centers in Hamburg and Kiel as described 13, 14. FISH was performed on fixed unstained slides of peripheral blood smears and tumor sections of FFPE material.

### Treatment

**Figure 1:**
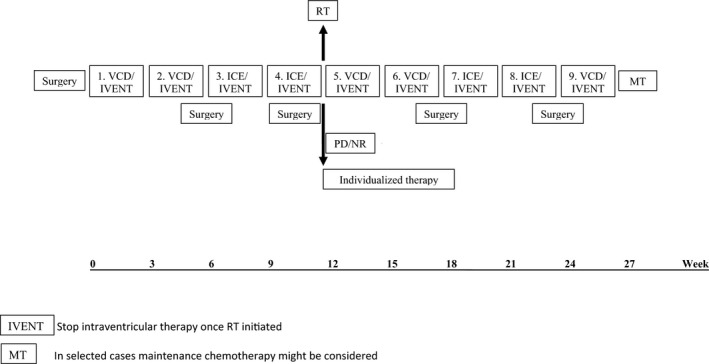
*Rhabdoid 2007* treatment recommendation for patients with AT/RT.

*Rhabdoid 2007* comprised postoperative chemotherapy with a total of nine courses including five alternating courses of vincristine, cyclophosphamide, doxorubicin (VCD) and four of ifosfamide, carboplatinum, etoposide (ICE) every 21 days (Fig. 1). Age‐adjusted intraventricular methotrexate (MTX) or triple chemotherapy (MTX/cytarabine/hydro‐cortisone) applied via Ommaya or Rickham reservoir was recommended with systemic chemotherapy until the start of radiotherapy. The suggested maintenance therapy (MT) consisted of eight cycles of trofosfamide/idarubicin (TI) and trofosfamide/etoposide (TE) every 3 weeks.

Conformal radiotherapy of the primary tumor region (encompassing the postsurgical tumor bed including any residual mass plus a 10–15 mm margin) was recommended at 54 Gy for patients over 18 months of age preferably after the 4th course of chemotherapy. In patients with CSF dissemination at diagnosis (M1 through M4 according to Chang) craniospinal radiotherapy with 24 Gy plus an additional boost to the primary tumor region and any central nervous system (CNS) deposits was recommended for ages 3 years and older. Circumscribed spinal deposits could be boosted up to 49.2 years. Innovative technologies such as IMRT or proton beam therapy were encouraged.

### Toxicity

Toxicity was assessed according to version 3.0. of the Common Terminology Criteria for Adverse Events. Reporting of serious adverse events (SAEs) to the registry office was requested but not monitored.

### Statistics

Overall (OS) and event‐free survival (EFS) were determined according to Kaplan–Meier estimates. OS was defined as the time from diagnosis until death of any cause or the last visit. EFS was defined as the time from diagnosis until first progression, relapse, death of any cause, or last contact.

The potential impact of prognostic factors on OS and EFS was analyzed as follows: congenital.

Kaplan–Meier analyses were performed for basic elements such as age and metastases, tumor location, extent of resection, germ‐line mutation (GLM), etc. Time‐dependent factors, for example, radiotherapy (RT), complete remission (CR), and MT were evaluated using Cox regression for time‐dependent covariates. *P*‐values were significant for *P* ≤ 0.05. Results were considered exploratory, not confirmatory. Adjustment for multiple testing was not performed. An overall significance level was not determined and could not be calculated due to small numbers.

## Results

### Clinical characteristics of patients with AT/RT

Thirty‐one patients were registered and treated according to *Rhabdoid 2007* (Table 1). This accounts for approximately 51% of all eligible cases from Germany during the corresponding time (2005–2009 total incidence *n *= 61). The median age at diagnosis of 12 females and 19 males was 20 months (range 0–120). Seventy‐four percent of patients were below 3 years at diagnosis (12 < 1 years, 11 = 1 – 3 years, 8 > 3 years). Three congenital cases were registered. One was symptomatic with visible multiple synchronous extracerebral tumors (thorax) in addition to an AT/RT at (no. 15). A second presented with a visible periorbital tumor plus AT/RT at birth (no. 19), the third demonstrated frequent vomiting and hydrocephalus leading to imaging (no. 28). Tumor localization was infratentorial (*n *= 13), supratentorial (*n *= 15), or encompassed both compartments (s.t. and i.t. *n *= 3). Imaging and CSF cytology revealed six patients with metastases at diagnosis. Four patients presented with CSF dissemination (M1), one patient with additional intracerebral metastases (M2), and one patient with soft tissue metastases (M4). Analysis of CSF was completed in 28 patients (no analysis in no. 3, 6, 15). Patients no. 3 and no. 6 were not stable enough for a lumbar puncture at diagnosis, no. 15 had multiple congenital synchronous tumors accompanied by soft tissue metastases (M4).

**Table 1:** Patient characteristics, therapeutic measures, and outcome.

No.	Age^a^	INI1	Germline mutation	Genetic analysis^b^	Diagnosis	Location	M‐stage^c^	Surgery	Chemotherapy^d^	Intraventricular therapy	HDCT	Radiotherapy	RT dose (GY)	Age at RT (months)	Maintenance therapy	Complete remission during therapy	Dead of disease	Survival (months)
1	4	neg	No	WT	AT/RT	Cerebellum	M0	Total	10	Yes	No	Local	54	15	No	Yes	Yes	–
2	56	neg	No	WT	AT/RT	Cerebral hemisphere	M0	Subtotal	8	Yes	No	Local	54	57	Yes	Yes	No	85
3	32	neg	n.a.	n.a.	AT/RT	Supratentorial n.s.	n.a.	Partial	6	No	Yes	No		–	No	No	Yes	–
4	5	neg	Yes	het del, dupl 838insT	AT/RT	Cerebellar hemisphere, IV. ventricle	M2	Total	5	Yes	No	No		–	No	No	Yes	–
5	7	neg	No	n.a.	AT/RT	Fossa cranii posterior	M0	Subtotal	4	Yes	No	No		–	No	No	Yes	–
6	29	neg	n.a.	WT	AT/RT	Tempo‐parietal	n.a.	Partial	3	No	No	No		–	No	No	Yes	–
7	14	neg	No	n.a.	AT/RT	Cerebellum	M0	Total	7	Yes	No	Local	54	21	Yes	Yes	No	75
8	23	neg	No	het del	AT/RT	Cerebral hemisphere frontal	M1	Subtotal	6	Yes	Yes	No		–	No	Yes	Yes	–
9	67	neg	No	1148delC ex9	AT/RT	Pineal gland, mesencephalon, thalamus	M0	Total	7	Yes	No	Local	54	70	Yes	Yes	No	77
10	11	neg	No	del chromosome 22	AT/RT	Cerebellum	M0	Subtotal	9	Yes	No	Local	54	19	Yes	Yes	No	95
11	19	neg	No	het del	AT/RT	Intracerebral	M0	Subtotal	9	Yes	No	Local	54	26	Yes	Yes	Yes	–
12	120	neg	No	het del, 1148delC ex9	AT/RT	Pineal gland, mesencephalon, III. ventricle	M0	Total	7	Yes	No	Local	54	122	Yes	Yes	No	79
13	38	neg	n.a.	n.a.	AT/RT	Cerebellar hemisphere, IV. ventricle	M0	Total	9	Yes	No	Local	54	44	No	Yes	No	66
14	5	neg	Yes	170delTG ex2	AT/RT	Cerebellum	M1	Subtotal	8	Yes	No	Local	54	12	Yes	Yes	Yes	–
15	0	neg	Yes	homo del	AT/RT+MRT	Cerebellopontine angle, thorax	M4	Biopsy	5	No	No	No		–	No	No	Yes	–
16	26	neg	n.a.	n.a.	AT/RT	Cerebral hemisphere	M0	Subtotal	9	Yes	No	Local	54	30	Yes	Yes	Yes	–
17	22	neg	n.a.	n.a.	AT/RT	Fossa cranii posterior	M0	Total	7	Yes	No	Local	54	29	Yes	Yes	No	79
18	10	neg	No	WT	AT/RT	Basal ganglia, lateral ventricle	M0	Partial	4	Yes	Yes	Local	54	15	No	Yes	No	96
19	0	neg	Yes	het und homo del	AT/RT+MRT	Orbi and periorbital, temporal head/neck	M0	Total	8	Yes	Yes	No		–	Yes	Yes	No	70
20	11	neg	n.a.	n.a.	AT/RT	Infratentorial n.s.	M0	Partial	8	Yes	Yes	Local	54	20	Yes	Yes	Yes	–
21	18	neg	No	n.a.	AT/RT	Cerebral hemisphere, mesencephalon, pons, medulla	M0	Partial	2	Yes	No	Local	54	25	Yes	No	Yes	–
22	6	neg	No	n.a.	AT/RT	Basal ganglia, pons	M0	Partial	6	Yes	No	Local	54	13	Yes	No	Yes	–
23	18	neg	No	Not evaluable	AT/RT	Mesencephalon, pons	M0	Total	9	Yes	No	Local	54	21	Yes	Yes	No	89
24	11	neg	No	511insC ex5	AT/RT	Fossa cranii posterior	M0	Subtotal	9	Yes	Yes	Local	54	29	Yes	Yes	No	66
25	88	neg	Yes	del, het dupl ex6	AT/RT	Fossa cranii posterior, cerebellum, medulla IV. ventricle	M0	Subtotal	11	Yes	No	Local	54	92	No	Yes	No	87
26	13	neg	No	WT	AT/RT	Cerebellum	M0	Partial	4	Yes	Yes	Local	54	21	No	Yes	No	60
27	49	neg	No	1148delC ex9	AT/RT	Cerebral hemisphere	M1	Partial	9	Yes	No	Craniospinal	54	52	Yes	Yes	No	60
28	0	pos	Yes	del ex17 SMARCA4	AT/RT	Cerebral hemisphere	M0	Subtotal	6	Yes	No	No		–	No	No	Yes	–
29	105	neg	No	homo del	AT/RT	Cerebral hemisphere	M0	Total	9	Yes	No	Local	54	110	No	Yes	No	64
30	60	neg	No	WT	AT/RT	Cerebral hemisphere	M1	Partial	8	Yes	Yes	Local + CSI	54 + 30	63	No	Yes	Yes	–
31	22	neg	No	del ex5	AT/RT	Cerebral hemisphere, basal ganglia	M0	Partial	9	Yes	No	Local	54	27	Yes	Yes	Yes	–

n.a., not analyzed; WT, wild‐type sequence; het, heterozygous; del, deletion; dupl, duplication; ins, insertion; ex, exon; homo, homozygous; n.s., not specified; neg, negative; pos, positive.

a Age at diagnosis in months.

b FISH, MLPA, sequencing in tumor.

c Metastases according to Chang classification.

d Courses.

The diagnosis was centrally confirmed on tumor tissue in all cases except one (no. 28) with an underlying *SMARCA4* GLM 4.

GLM were detected in six out of 21 evaluated patients. Five mutations affected the *SMARCB1* and one the *SMARCA4* locus (Table 2). All three patients with congenital tumors displayed a GLM (no. 15, 19, and 28).

**Table 2:** Safety – serious adverse events recorded while treatment according to the “Rhabdoid 2007” protocol.

Serious adverse events (13 SAE in 12/31 patients)	*n*
Necrotizing leukoencephalopathy (causing therapy discontinuation)	1
Radiogenic gliosis	1
Sepsis (Ommaya infection *n*=2, post‐op *n*=1)	7
Recurrent infections (causing therapy discontinuation)	1
Coxitis with candida	1
Pneumonia (causing discontinuation of maintenance)	1
Shunt insufficiency causing death (not due to disease)	1

### Response to chemotherapy

All patients received chemotherapy according to *Rhabdoid 2007* (Fig. 1). Nine of them completed the recommended schedule of nine courses. Two patients received additional courses (no. 1, no. 25). The reasons were (no. 1) bridging until initiation of radiotherapy (RT) and (no. 25) combination with RT for expected synergistic effects. Seven patients received HDCT following induction (after a different number of courses [range 4–8]), and one patient (no. 24) after completed chemotherapy (9 courses).

Thirteen patients received 2–8 courses of conventional chemotherapy courses. Seven patients discontinued chemotherapy due to disease progression, two due to toxicities (no. 7, 9), and one due to shunt insufficiency (no. 22). Three patients had started therapy according to protocols for different entities until definitive reference diagnosis was available (no. 2, 12, 17).

Twenty‐eight patients received strict intraventricular chemotherapy. Twenty‐three of these had no sign of M+ disease in the CSF, five patients were positive (no. 4, 8, 14, 27, 30). Reasons for not applying intraventricular therapy were initiation of concomitant radiotherapy, insufficient circulation of CSF and/or CNS infections.

MT was applied to 17 patients, 14 patients did not receive it due to early disease progression or decision of the treating physician.

### Radiotherapeutic approach

Radiotherapy was applied locally to the primary tumor region in 23 of 31 patients. Six patients did not receive RT due to early disease progression, one patient due to age <18 months (no. 19), and one patient (no. 28) due to necrotizing leukoencephalopathy. The age of patients at the time of radiotherapy ranged from 1 to 10 years (Table 1). Fifteen patients were younger than 36 months old, and four (no. 1, 14, 18, 22) younger than 18 months at RT (15, 12, 15, 13 months). Two of the four patients died from progressive disease and one following VP‐shunt insufficiency.

Of 23 patients who had received radiotherapy, 20 were diagnosed with M_0_ and 3 with M_1_ disease (no. 14, 27, 30). One (no. 14) patient with M_1_ disease received only local radiotherapy, another patient (no. 27) craniospinal, and the third patient (no. 30) craniospinal and boost radiotherapy. One had a relapse while still on therapy, the second is alive 5 years from diagnosis and the third died 6 years following diagnosis. Three of six patients with M+ (no. 4, 8, 15) did not receive radiotherapy due to progression.

Proton beam therapy to the tumor region was applied to 4 patients (no. 7, 11, 14, 30) at 21, 26, 12, 63 months of age.

### Response to therapy and outcome

A CR was achieved in 23/31 patients. Four patients presented with M1 at diagnosis (no. 8, 14, 27, 30). In 10 CR was achieved by neurosurgical resection (in no. 26 after second look surgery) and in 13 by additional chemo‐and radiotherapy. Five patients achieved CR after two courses of induction, two after three courses, one after four courses, one after five courses, four patients thereafter.

Follow‐up of survivors comprises 61–96 months FUP (follow‐up period) with a median of 77 months. Fifteen patients survived, another 15 died due to disease progression/relapse and one (no. 22) following shunt insufficiency without signs of relapse on imaging. Only one patient with metastatic disease survived (no. 27), but also one with synchronous tumor and GLM (no. 19). Ten patients received a total resection and were in CR; another one (no. 26) after second look surgery. One of these relapsed (no. 1) and one (no. 4) progressed despite total resection and died. Among 20 patients with residual disease following surgery one (no. 22) died following shunt insufficiency without relapse, six eventually demonstrated progressive disease. Of 13 patients who achieved CR on chemotherapy seven died due to relapse 1–23 months after the end of therapy. Whenever feasible RT was performed as a rescue measure.

**Figure 2:**
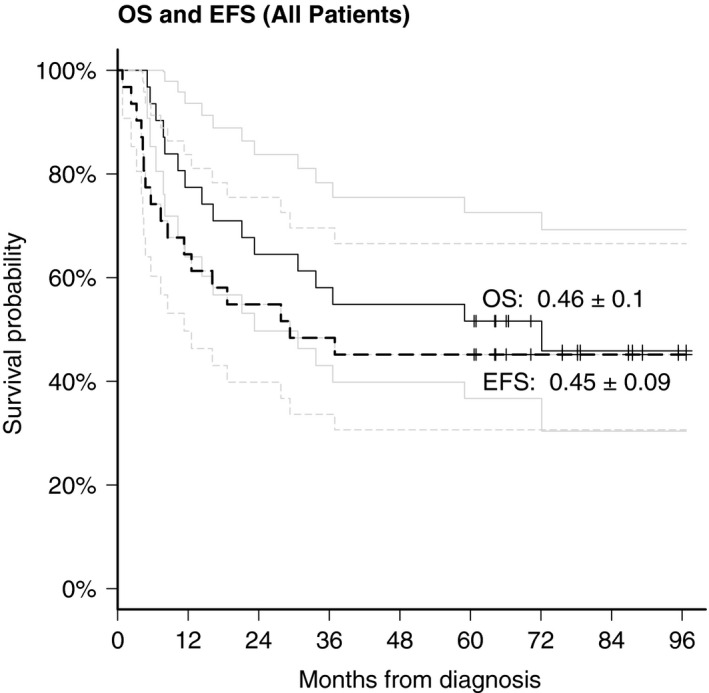
Overall survival and event‐free survival of the Rhabdoid 2007 cohort *n* = 23.

Six‐year overall and event‐free survival rates are 46% (±0.10) and 45% (±0.09) resp. (Fig. 2). The latest relapse was 37 months following diagnosis. This patient (no. 11) succumbed 5 years after initial diagnosis from relapse despite having achieved a CR to initial therapy. The latest death thus far occurred 72 months from diagnosis (no. 31).

### Prognostic factors in the cohort Rhabdoid 2007

**Figure 3:**
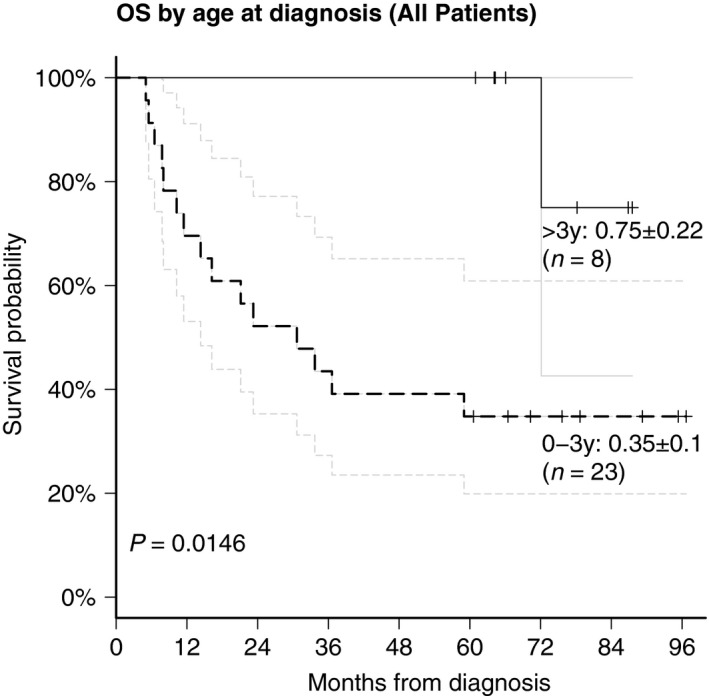
Statistical analysis of prognostic factors: influence of age on survival.

**Figure 4:**
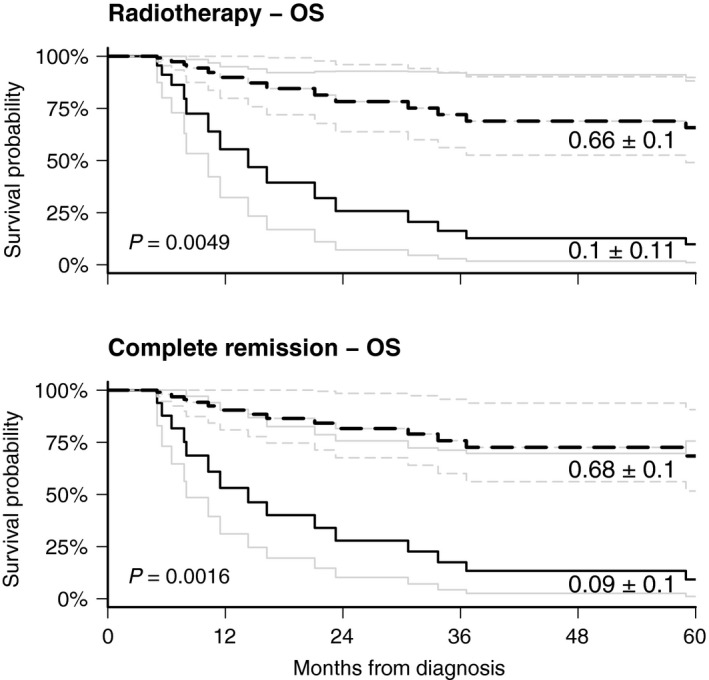
Influence of radiation therapy and achievement of complete remission on survival, cox model with time‐dependent variates.

The Kaplan–Meier estimates for factors influencing OS demonstrated a significant prognostic benefit for age > 3y at diagnosis (*P* < 0.015) (Fig. 3). Of the eight patients who were older than 3 years at diagnosis only one relapsed and died (no. 30), seven are alive 61–85 months following diagnosis. Cox regression analysis for time‐dependent covariates demonstrated a significant impact of radiotherapy (*P* < 0.005) and achievement of a CR (*P* < 0.002) on OS (Fig. 4).

Presence of metastases at diagnosis, complete resection, and GLM did not influence outcome in this series. Intraventricular MTX or MT did not show any beneficial effect on OS.

### Patterns of failure in AT/RT

Sixteen of 31 patients experienced treatment failure and all but one (death due to ventriculo‐peritoneal shunt insufficiency) died due to disease. The median time to progression or relapse was 6.5 months (range 1–37 months). In nine patients early progression or relapse occurred while on therapy. The latest relapse with intracranial metastases outside the proton radiotherapy field was recorded 37 months after diagnosis (no. 11). Pattern of progression/relapse included seven patients with progress disease, six local, and two combined (local + distant) failures.

### Toxicities of therapy according to Rhabdoid 2007

A total of 13 SAEs were reported in 12/31 patients (Table 2). One treatment‐related death due to insufficiency of a VP‐shunt and subsequent herniation of the brain was noted (no. 22). Three SAE led to therapy discontinuation due to pneumonia, recurrent infections, or necrotizing leukoencephalopathy associated with intraventricular MTX (no. 9, 23, 28).

## Discussion

### Challenges in the care of patients with AT/RT

The prognosis of patients with AT/RT remains dismal despite intensive multimodal therapy. Reinhard et al. reported a 5‐year OS of 29% for 25 patients with AT/RT 5. Dufour et al. presented data of 58 AT/RT patients treated from 1998 to 2008 with a median OS of 9 months 7. Comparable unfavorable results of a median OS of 13.5 months were reported by the Canadian Pediatric Brain Consortium 16. Recently, data of a first clinical trial in 20 patients with AT/RT indicated a promising 2‐year OS of 70%, but no follow‐up data are available yet 8. It appears that 5‐year OS rates around 40–50% are achievable (S. N. Chi, pers. comm.). Rather provocative data have been published by Slavc et al. with OS rates of 100% in nine patients. Independent confirmation is currently missing 15.

Reasons for the rather limited data are the rare incidence, high vulnerability of very young infants and children, localization in critical anatomical structures, potential for synchronous tumors and likelihood for chemoresistance. Thus, rhabdoid tumors are far from being ideal subjects for controlled clinical trials. Interest by pharmaceutical companies in performing drug trials with novel compounds has been sparse due to the inherent risk associated with mentioned factors.

The highest level of evidence will certainly be obtained by controlled clinical trials if possible evaluating research questions in a randomized fashion. However, children with rare diseases and a risk profile, which makes a controlled trial extremely difficult, do have the same right for a standardized treatment with the best available scientific evidence of activity.

### The need for comprehensive data sets in AT/RT

To circumvent these obstacles German oncologists and other investigators around the world have chosen to recruit patients into “registry studies.” These competence structures offer a system of high‐quality reference diagnostics and expert counseling for diagnosis and therapy. The resulting data are inferior in quality to a controlled clinical trial, but superior to case series and individual reports. The reference system established within the GPOH has over the last decades achieved a near 99% coverage of affected children and is employed throughout German speaking countries.

Results of only one prospective clinical trial have been published in AT/RT 8. At the time of initiation of the EU‐RHAB registry in 2005 a standard of therapy had not been consented. As a European group we therefore chose the means of a registry. It has by now been joined by several European countries and has continued collecting data for almost 10 years. This modality was meant to bridge the gap between case series and the start of a clinical trial not to lose valuable data. The EU‐RHAB database allows conclusions on prognostic factors, course of disease and treatment modalities, onto which together with the diagnostic reference structure future phase I/II clinical trials will be built.

As expected there were deviations from to the protocol according to clinical conditions and individual preferences. The resulting groups with small numbers may not provide statistical significance and the results are complex to interpret. Despite these facts *Rhabdoid 2007* therapy resulted in 6‐year OS and EFS rates of 46% (±0.10) and 45% (±0.09). This is an acceptable outcome when compared with published data and is based on a relevant follow‐up period of median of 6 years (5–8 years).

### The prognostic variables determining outcome in AT/RT

Results from the registry study *Rhabdoid 2007* provide information of prognostic factors in accordance with those previously described such as age at diagnosis 6, 7, 17. In our cohort seven of eight patients older than 3 years at diagnosis are long‐term survivors. Furthermore, Cox regression analysis for time‐dependent covariates demonstrated that a CR and the application of radiotherapy were positive prognostic factors. As described by Chi et al. CR by primary tumor therapy seems to be an important prognostic factor for survival 8, 18. Our analyses confirmed this finding. In the *current* cohort all surviving patients (*n *= 15) had achieved a CR. Nevertheless, of 23 who had achieved CR eight patients experienced late relapses and died.

Another negative prognostic factor appears to be the presence of GLM 19. Affected patients generally have a rather poor prognosis with a 2‐year survival of 29% 19. However, detailed data have recently been reported on four patients, including two of ours (no. 19, 25), with a predisposition to rhabdoid tumors and EFS of 5 and 7 years from diagnosis 14. In our cohort, no influence of GLM on OS was detected. However, only six patients had GLM, two of them are long‐term survivors (no. 19, 25). Two further cases in our cohort showed an unexpected long survival despite unfavorable prognostic factors. One patient with M1 is still alive more than 5 years from diagnosis (no. 27) and another patient survived recurrence for 22 months until death of disease (no. 11). These cases indicate that even in unfavorable situations a therapeutic attempt is justified and could result in an unexpected survival time.

In our cohort there was a high likelihood of survival once patients reached 3 years from diagnosis without relapse. The latest relapse occurred 37 months after diagnosis (no. 11) and the follow‐up in our cohort ranged from 60 up to 96 months. Still, the literature knows of a case of late intracranial recurrence 5.5 years following diagnosis 20. The majority of our patients experiencing treatment failure progressed on therapy, which indicates chemotherapy resistance. Especially those patients will most likely not benefit from further intensification of treatment but rather from agents targeting specific pathways. Since all relapsed patients in our cohort died, there is an evident need for relapse trials with innovative concepts in AT/RT.

### The potential role of radiotherapy in the treatment of AT/RT

Radiotherapy has been deemed to be a significant positive prognostic factor on retrospective analyses 17. The NCI identified RT as an independent factor for survival in retrospective analysis of 144 children with AT/RT 21. It was concluded that RT could be of significant benefit particularly when looking at very young children <3 years. For our cohort the multivariate, time‐dependent statistical analysis demonstrated a significant beneficial effect of RT on OS and 60% of irradiated patients were <3 years. Assessment of the true value of RT in our cohort is complicated by the fact that most patients who did not receive RT were below 1 year of age at diagnosis and experienced rapid progressive disease. As a majority of progressions occurred on chemotherapy it deserves discussion whether RT as a rescue measure should be given earlier, for example, even to patients below 18 months of age 22. Late effects of RT in young children are, however, significant and have to be considered when tailoring treatment concepts 23. Increasingly, modern technologies like proton beam therapy are used and recent reports demonstrate both good feasibility and promising local control rates 24, 25.

The role of single therapeutic components in the concept of multimodal therapy such as HDCT, intraventricular MTX and MT remains to be clarified. In our cohort the analysis of these three modalities showed no beneficial effect on OS. Data from 19 patients with HDCT of the EU‐RHAB registry have been published 10 but due to the retrospective nature and a heterogeneously treated group with small patient numbers, no definitive conclusion about the effect of HDCT as primary therapy for AT/RT may be drawn.

### Feasibility of intensive multimodal therapy in children with AT/RT

Despite a major proportion of >70% of patients below the age of 3 years in our cohort, including 35% infants, no toxic death due to chemotherapy or RT occurred. One treatment‐related death was reported following VP‐shunt insufficiency. In three cases (no. 7, 9, 22) early termination of chemotherapy was necessary due to complications of chemotherapy and intraventricular MTX. With respect to the young age of the patient population and treatment intensity, the recommended treatment of *Rhabdoid 2007* showed feasibility and safety. Still, a potential for underreporting of adverse events due to the voluntary character in the framework of the registry has to be acknowledged.

Possible late sequelae which are recognized after intensive therapy for other malignancies in early childhood 26 will be investigated more detailed in AT/RT patients in the future.

## Conclusion

Our results demonstrate that intensive multimodality treatment is feasible, safe and efficient even in very young patients. This supports the concept of EU‐RHAB as the basis for data analysis but also for future clinical trials in first line and relapse therapies. The implementation of a phase I/II study for AT/RT and other rhabdoid tumors in the frame of the established registry structures of EU‐RHAB is essential for further improvement of survival. Recently detected pathogenic mechanisms such as epigenetic modulation should give clues which compounds to choose for future innovative trials 27.

## Conflict of Interest

None of the authors have any conflict of interest to declare.

## References

[1] Judkins, A. R. , J. Mauger , A. Ht , L. B. Rorke , and J. A. Biegel . 2004 Immunohistochemical analysis of hSNF5/INI1 in pediatric CNS neoplasms. Am. J. Surg. Pathol. 28:644–650. 15105654 10.1097/00000478-200405000-00013

[2] Hasselblatt, M. , S. Isken , A. Linge , K. Eikmeier , A. Jeibmann , F. Oyen , et al. 2013 High‐resolution genomic analysis suggests the absence of recurrent genomic alterations other than SMARCB1 aberrations in atypical teratoid/rhabdoid tumors. Genes Chromosom. Cancer 52:185–190. 23074045 10.1002/gcc.22018

[3] Kieran, M. W. , C. W. Roberts , S. N. Chi , K. L. Ligon , B. E. Rich , L. E. Macconaill , et al. 2012 Absence of oncogenic canonical pathway mutations in aggressive pediatric rhabdoid tumors. Pediatr. Blood Cancer 59:1155–1157. 22997201 10.1002/pbc.24315 PMC3538080

[4] Hasselblatt, M. , I. Nagel , F. Oyen , K. Bartelheim , R. B. Russell , U. Schüller , et al. 2014 SMARCA4‐mutated atypical teratoid/rhabdoid tumors are associated with inherited germline alterations and poor prognosis. Acta. Neuropathol. 128:453–456. 25060813 10.1007/s00401-014-1323-x

[5] Reinhard, H. , J. Reinert , R. Beier , R. Furtwängler , M. Alkasser , S. Rutkowski , et al. 2008 Rhabdoid tumors in children: prognostic factors in 70 patients diagnosed in Germany. Oncol. Rep. 19:819–823. 18288421

[6] Tekautz, T. M. , C. E. Fuller , S. Blaney , M. Fouladi , A. Broniscer , T. E. Merchant , et al. 2005 Atypical teratoid/rhabdoid tumors (ATRT): improved survival in children 3 years of age and older with radiation therapy and high‐dose alkylator‐based chemotherapy. J. Clin. Oncol. 23:1491–1499. 15735125 10.1200/JCO.2005.05.187

[7] Dufour, C. , A. Beaugrand , M. C. Le Deley , F. Bourdeaut , N. André , P. Leblond , et al. 2012 Clinicopathologic prognostic factors in childhood atypical teratoid and rhabdoid tumor of the central nervous system: a multicenter study. Cancer 118:3812–3821. 22180295 10.1002/cncr.26684

[8] Chi, S. N. , M. A. Zimmerman , X. Yao , K. J. Cohen , P. Burger , J. A. Biegel , et al. 2009 Intensive multimodality treatment for children with newly diagnosed CNS atypical teratoid rhabdoid tumor. J. Clin. Oncol. 27:385–389. 19064966 10.1200/JCO.2008.18.7724 PMC2645855

[9] Hilden, J. M. , S. Meerbaum , P. Burger , J. Finlay , A. Janss , B. W. Scheithauer , et al. 2004 Central nervous system atypical teratoid/rhabdoid tumor: results of therapy in children enrolled in a registry. J. Clin. Oncol. 22:2877–2884. 15254056 10.1200/JCO.2004.07.073

[10] Benesch, M. , K. Bartelheim , G. Fleischhack , B. Gruhn , P. G. Schlegel , O. Witt , et al. 2014 High‐dose chemotherapy (HDCT) with auto‐SCT in children with atypical teratoid/rhabdoid tumors (AT/RT): a report from the European Rhabdoid Registry (EU‐RHAB). Bone Marrow Transplant. 49:370–375. 24419520 10.1038/bmt.2013.208

[11] Chang, C. H. , E. M. Housepian , and C. Herbert Jr . 1969 An operative staging system and a megavoltage radiotherapeutic technic for cerebellar medulloblastomas. Radiology 93:1351–1359. 4983156 10.1148/93.6.1351

[12] Warmuth‐Metz, M. , B. Bison , and S. Leykamm . 2009 Neuroradiologic review in pediatric brain tumor studies. Klin. Neuroradiol. 21:21. 10.1007/s00062-009-9029-5 19936570

[13] Kordes, U. , S. Gesk , M. C. Frühwald , N. Graf , I. Leuschner , M. Hasselblatt , et al. 2010 Clinical and molecular features in patients with atypical teratoid rhabdoid tumor or malignant tumor. Genes Chromosom. Cancer 49:176–181. 19902524 10.1002/gcc.20729

[14] Kordes, U. , K. Bartelheim , P. Modena , M. Massimino , V. Biassoni , H. Reinhard , et al. 2014 Favorable outcome of patients affected by rhabdoid tumors due to rhabdoid tumor predisposition syndrome (RTPS). Pediatr. Blood Cancer 61:919–921. 24123847 10.1002/pbc.24793

[15] Slavc, I. , M. Chocholous , U. Leiss , C. Haberler , A. Peyrl , A. A. Azizi , et al. 2014 Atypical teratoid rhabdoid tumor: improved long‐term survival with an intensive multimodal therapy and delayed radiotherapy. The Medical University of Vienna Experience 1992–2012. Cancer Med. 3:91–100. 24402832 10.1002/cam4.161 PMC3930393

[16] Lafay‐Cousin, L. , C. Hawkins , A. S. Carret , D. Johnston , S. Zelcer , B. Wilson , et al. 2012 Central nervous system atypical teratoid rhabdoid tumours: the Canadian Paediatric Brain Tumour Consortium experience. Eur. J. Cancer 48:353–359. 22023887 10.1016/j.ejca.2011.09.005

[17] Sultan, I. , I. Qaddoumi , C. Rodriguez‐Galindo , A. A. Nassan , K. Ghandour , and M. Al‐Hussaini . 2010 Age, stage, and radiotherapy, but not primary tumor site, affects the outcome of patients with malignant rhabdoid tumors. Pediatr. Blood Cancer 54:35–40. 19798737 10.1002/pbc.22285

[18] von Hoff, K. , B. Hinkes , E. Dannenmann‐Stern , A. O. von Bueren , M. Warmuth‐Metz , N. Soerensen , et al. 2011 Frequency, risk‐factors and survival of children with atypical teratoid rhabdoid tumors (AT/RT) of the CNS diagnosed between 1988 and 2004, and registered to the German HIT database. Pediatr. Blood Cancer 57:978–985. 21796761 10.1002/pbc.23236

[19] Bourdeaut, F. , D. Lequin , L. Brugieres , S. Reynaud , C. Dufour , F. Doz , et al. 2011 Frequent hSNF5/INI1 germline mutations in patients with rhabdoid tumor. Clin. Cancer Res. 17:31–38. 21208904 10.1158/1078-0432.CCR-10-1795

[20] Modena, P. , I. Sardi , M. Brenca , L. Giunti , A. M. Buccoliero , B. Pollo , et al. 2013 Case report: long‐term survival of an infant syndromic patient affected by atypical teratoid‐rhabdoid tumor. BMC Cancer 13:100. 23510391 10.1186/1471-2407-13-100 PMC3600022

[21] Buscariollo, D. L. , H. S. Park , K. B. Roberts , and J. B. Yu . 2012 Survival outcomes in atypical teratoid rhabdoid tumors for patients undergoing radiotherapy in a surveillance, epidemiology, and end results analysis. Cancer 118:4212–4219. 22213196 10.1002/cncr.27373

[22] Seeringer, A. , K. Bartelheim , K. Kerl , M. Hasselblatt , I. Leuschner , S. Rutkowski , et al. 2014 Feasibility of intensive multimodal therapy in infants affected by rhabdoid tumors ‐ experience of the EU‐RHAB registry. Klin. Padiatr. 226:143–148. 24633978 10.1055/s-0034-1368719

[23] Hasan, A. , M. Palumbo , J. Atkinson , A. S. Carret , J. P. Farmer , J. Montes , et al. 2011 Treatment‐related morbidity in atypical teratoid/rhabdoid tumor: multifocal necrotizing leukoencephalopathy. Pediatr. Neurosurg. 47:7–14. 21613772 10.1159/000323412

[24] Weber, D. C. , C. Ares , R. Malyapa , F. Albertini , G. Calaminus , U. Kliebsch , et al. 2015 Tumor control and QoL outcomes of very young children with atypical teratoid/rhabdoid tumor treated with focal only chemo‐radiation therapy using pencil beam scanning proton therapy. J. Neurooncol. 121:389–397. 25362544 10.1007/s11060-014-1648-2

[25] Haskins, C. P. , B. Jyoti , M. Hines , V. Simoneaux , and J. C. Buchsbaum . 2015 Single center results following proton beam therapy in children with atypical teratoid rhabdoid tumors of the central nervous system. Int. J. Particle Ther. 2:1–10.

[26] Robinson, K. E. , C. E. Fraley , M. M. Pearson , J. F. Kuttesch Jr , and B. E. Compas . 2013 Neurocognitive late effects of pediatric brain tumors of the posterior fossa: a quantitative review. JINS 19:44–53. 23095276 10.1017/S1355617712000987

[27] Kerl, K. , T. Holsten , and M. C. Fruhwald . 2013 Rhabdoid tumors: clinical approaches and molecular targets for innovative therapy. Pediatr. Hematol. Oncol. 30:587–604. 23848359 10.3109/08880018.2013.791737

